# Examining human-animal interactions and their effect on multidimensional frailty in later life: a scoping review

**DOI:** 10.3389/fpubh.2023.1214127

**Published:** 2023-06-21

**Authors:** Ashley Taeckens, Mary Corcoran, Kaipeng Wang, Kevin N. Morris

**Affiliations:** ^1^Institute for Human-Animal Connection, Graduate School of Social Work, University of Denver, Denver, CO, United States; ^2^Graduate School of Social Work, University of Denver, Denver, CO, United States

**Keywords:** frailty, human-animal interaction (HAI), pet ownership, older adults, health domains, quality of life

## Abstract

This scoping review sought to compile outcomes associated with any human-animal interaction study regarding adults aged 50 and older in any living context and concerning a multidimensional (i.e., physical, psychological, cognitive, and social) perspective of frailty. Despite our best attempts at incorporating the broadest inclusion criteria possible, only four articles were relevant to this review. Participants across the included studies were rural, community-dwelling Japanese or Chinese individuals aged 60 years and older. Thematic analysis of reported results includes dog ownership as a protective factor regarding frailty, the interconnected health effects of pet ownership, and increased meaning and purpose through pet ownership implications. More research is needed globally to determine how human-animal interactions may moderate frailty comprehensively, as well as the efficacy and appropriateness of these interactions or interventions in older adult populations and across cultural boundaries.

## 1. Introduction

Physical, psychological, social, and cognitive health trajectories have been widely studied, but these concepts as domains of frailty are rarely assessed concurrently. Frailty can hinder well-being and quality of life (QOL), yet the lack of a universal definition (1) of this health challenge impedes assessment of its prevalence and identification of appropriate interventions. Some have focused their work on physical (2, 3) or cognitive elements of frailty (4), but significant gaps remain regarding an exhaustive understanding of this phenomenon. A comprehensive definition may yield better understandings of how frailty occurs, within what health domains, and steps to prevent or mitigate it to uphold well-being and QOL over time.

Cumulative deficit perspectives have been proposed but rarely incorporate a comprehensive list of variables to account for frailty risks. Like cognitive health trajectories (5), frailty risk often increases gradually over time (6) with strong positive correlations existing between risk and old age. Frailty is also associated with decreased autonomy, which can result in poorer physical and psychological health for older adults (6) *via* hindered positive affect and feelings of self-efficacy (7).

As global demographics continue to age and the pertinence of independence endures, we call for researchers, practitioners, and policymakers to effectively identify and intervene against frailty risks. One such possible intervention is human-animal interaction (HAI). Interactions with the same animal over time can reduce cardiovascular activity and cortisol levels (8), and may also contribute to stress reduction, perceptions of adequate social support (9), and improved mobility (10). Existing reviews suggest potential associations between dog ownership, social contact, and well-being (11). Moreover, improved symptoms of depression, anxiety, cognitive impairment, and even dementia are reportedly associated with HAIs (12).

Human-animal interactions (HAIs) constitute the many modalities that facilitate exchanges between humans and other animals. Animal-assisted therapy is a type of HAI intervention where “an animal that meets specific criteria is an integral part of the treatment process” (13, p. 34). These therapeutic interactions require service delivery by a professional with specialized expertise and in specific settings (13). Alternatively, animal-assisted activities “[provide] opportunities for motivational…and/or therapeutic benefits to enhance [QOL]” (13, p. 34), which can be delivered in different settings and with varying levels of expertise. Other forms of HAI include visiting animals, service animals, and emotional support animals. Pet ownership (PO) is the primary HAI driving this work and includes interactions with domesticated pets and farm animals.

We aimed to compile outcomes on any HAI study with older adults regarding a multidimensional perspective of frailty. We defined frailty as a dynamic process involving both losses and increased vulnerability regarding one’s psychological, physical, cognitive, and social functioning that are correlated with chronological age. The guiding research question was, “What is known about HAI studies available to 50+ adults regarding their effect on frailty?.” To our knowledge, a relevant scoping review does not yet exist. A related systematic review has been conducted and is included here (14) but incorporated superfluous exclusion criteria that necessitated this expanded literature review (e.g., only searched PubMed and Google Scholar for articles published between 2000 and 2020; excluded randomized controlled trials, reviews, editorials, dissertations, and conference abstracts; only included healthy participants).

## 2. Methods

### 2.1. Search strategy

**Figure 1:**
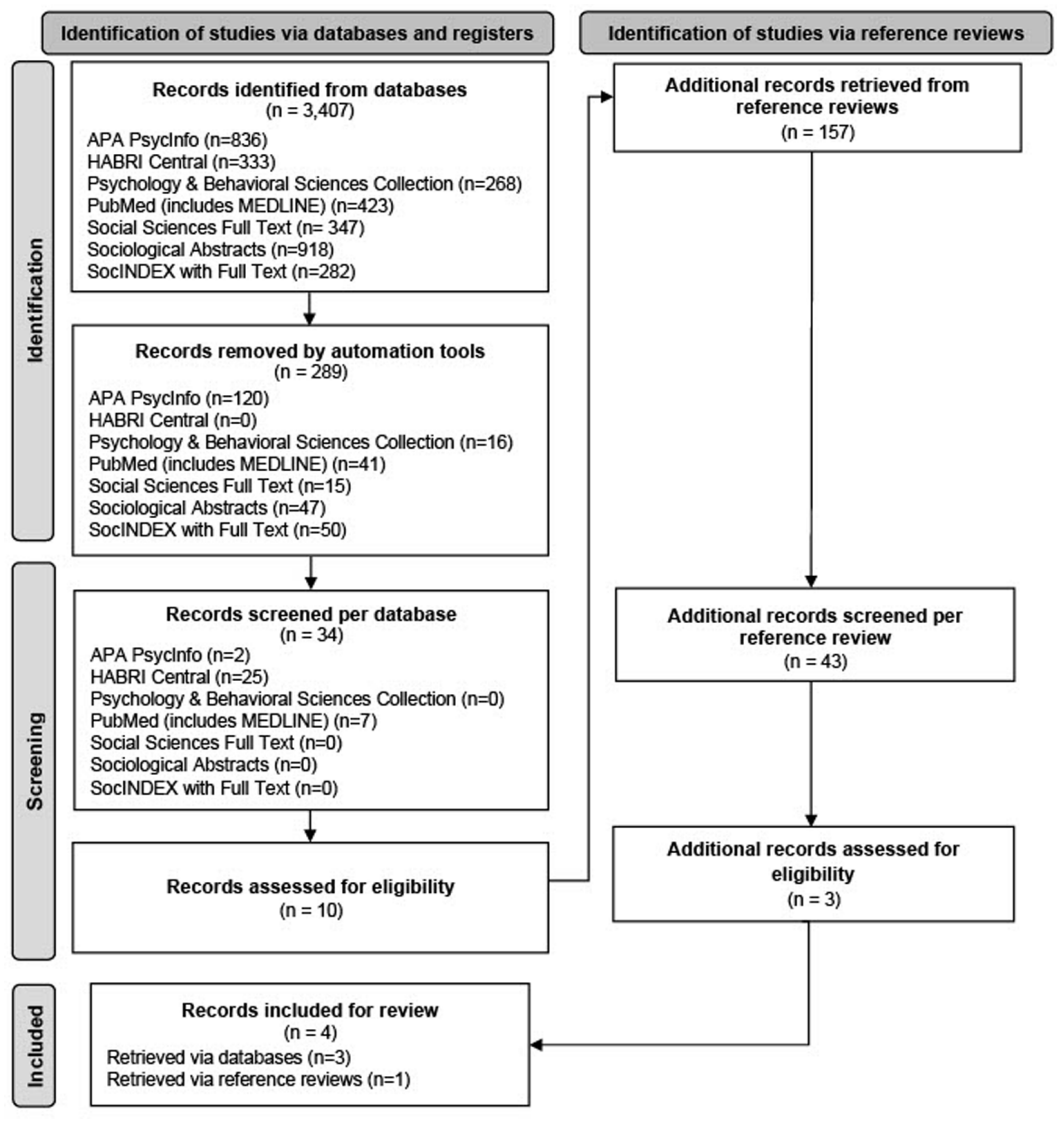
PRISMA Flow diagram of the scoping review search and selection process.

This review followed established guidelines (15) and the Preferred Reporting Items for Systematic Reviews and Meta-Analyses extension for scoping reviews (PRISMA-ScR) (16). We consulted with an expert librarian from our university in May 2022 for feedback on search terms, truncation, Boolean phrasing, and databases best suited for this review. The final search terms are included in Supplementary Table S1a. Six databases were searched in June 2022 using EBSCOhost (APA PsycInfo, PubMed [includes MEDLINE], Sociological Abstracts, SocINDEX with Full Text, Social Science Full Text, and Psychology and Behavioral Sciences Collection) as well as one publicly available platform (HABRI Central). Google Scholar and Web of Science Collection searches were attempted but inaccessible due to imposed character limits. The reference lists of assessed articles were also screened for inclusion. Our PRISMA-ScR Flow diagram is provided in Figure 1.

### 2.2. Inclusion and exclusion criteria

Articles were required to provide a frailty definition and relevant instrumentation or otherwise consider it an outcome. Those that only addressed human-animal observations (i.e., aviaries, fish tanks) were excluded due to the notable emphasis on physical conceptualizations and measurements of frailty in the literature (17). All forms of academic manuscripts published in English were considered. Participants must be 50+ but could have reported any form of housing or living arrangement (e.g., multigenerational; retirement community residents), any health status (i.e., healthy, unhealthy) or any type of physical, cognitive, or psychiatric diagnosis or comorbidity (e.g., cognitive impairment; mental illnesses).

### 2.3. Study selection

After downloading full texts to Zotero for screening, core data was extracted (e.g., frailty instrumentation; animal species) and further assessed for eligibility. From the articles included for review *via* database searches, the first author reviewed each reference list and screened titles and abstracts to identify other relevant articles. Reported key findings and implications were charted and thereupon analyzed for inductive thematic analysis.

## 3. Results

### 3.1. Search outcomes

Overall, 3,407 sources were retrieved. After 289 duplicates were automatically removed and titles and abstracts of the remaining 3,118 unique articles were assessed, the first two authors independently read through 34 full texts to determine eligibility. Thereafter, 43 articles identified *via* reference reviews were screened and three were assessed for eligibility. Any inclusion uncertainty was resolved through discussion among the first two authors. A total of four articles are included: two were retrieved from PubMed (includes MED-LINE) (4, 14), one from HABRI (Human Animal Bond Research Institute) Central (18), and one from reference reviews (19).

### 3.2. Study characteristics

#### 3.2.1. Settings and participants

All four studies focused on the effects of self-selected pet ownership—predominantly dogs and cats—among community-dwelling individuals. Participants across the included studies were predominantly women, lived in East Asia (*n* = 3 Japan, *n* = 1 China), and averaged 60+ years. Additional characteristics (e.g., household size) were inconsistently collected.

Of the three original research articles, one interviewed older adults (*N* = 2,638) in rural environments who experienced the “social loss” of either divorce or widowhood to determine whether PO influenced cognitive frailty trajectories (4), but excluded individuals 85+, those not fluent in Chinese, and with dementia or psychiatric diagnoses. Two studies surveyed 65+ adults on the experience of dog or cat ownership (*N* = 11,233) (18, 19), with one requiring participants to be non-frail (*N* = 6,197). For additional attributes of included publications, see Supplementary Table S1b.

#### 3.2.2. Study design, procedures, and materials

Two of the included studies were cross-sectional (4, 19), one was cross-sequential (18), and one was a systematic review (14). Of the original research articles, two utilized self-administered mail surveys for data collection (18, 19) and one used structured face-to-face interviews (4). Each article asked a variation of the dichotomous question, “Do you live with a pet?” (4, 18, 19). Two asked whether one lived with a pet currently, in the past, or never (18, 19), whereas another asked if one *owned* a pet (4). For those who either currently or had lived with a pet, two articles asked whether it was a “dog, cat, or other” (18, 19), and one asked whether the pet was a “dog, cat, bird, [or] other” (4). One article did not analyze data by way of PO type, nor was it concerned with past PO (4).

#### 3.2.3. Defining frailty

Various conceptualizations of frailty were uncovered. Two of the four studies defined frailty using outcome measures (14, 19). One article specifically concerned with “incident frailty” conceptualized it as a combination of “physical frailty” and “social frailty” (18). Another homed into the effects of PO on one’s “cognitive frailty**,”** or the combination of physical frailty and cognitive impairment (4). Additional concepts, like “psychological frailty” and environmental health (20), were encountered but did not meet inclusion criteria (21). Notably, only one article spoke to the concept of frailty as potentially reversible (4).

#### 3.2.4. Measuring frailty

Three articles (14, 18, 19) assessed frailty using the Kaigo-Yobo Checklist (CL15) (22), where a score of four or more (≥4) typically indicates frailty. However, one of those studies reported “a score *higher* than [four] was defined as frailty” (>4) (19, p. 3), deriving confusion as to what the appropriate cutoff score is. One article included in the reported systematic review measured frailty using a modification of the Cardiovascular Health Study (23), which was foundational to the development of the frailty phenotype (3). Another measured “cognitive frailty” by defining it as the presence of both cognitive impairment and a score of ≥3 on the frailty phenotype (4). This phenotype is a five-item scale to determine frailty risk through a combination of physical domain variables (shrinking, weakness, exhaustion, slowness, low physical activity level). Respectively, frailty is defined as a clinical syndrome made up of the presence of ≥3 of the included criteria, with the risk of frailty defined as a score of one or two (3).

#### 3.2.5. Additional measures

The Mini-Mental State Examination (24) and frailty phenotype (3) were used to evaluate the presence of cognitive frailty (4). All additional measures exclusively pertained to covariates (e.g., functional impairment).

### 3.3. Reported results of included studies

The following results are organized by article and in chronological order.

Survey responses of Ota Genki Senior Project participants (*N* = 11,233) were analyzed regarding their experiences of “dog/cat ownership” to examine physical, psychological, and social functioning among current, past, and never pet owners (19). While no significant differences in frailty (i.e., score > 4) were found among current, past, and never owners through this scoring mechanism, compared to never owners, past and current ownership was associated with benefits in physical, social, and psychological functioning domains (19). This work suggests that never owning a dog/cat is associated with lower levels of walking, lesser degrees of light physical activity, and poorer social functioning (i.e., feelings of trust in neighbors) (*p* < 0.001) (19). Finally, certain health characteristics were reported as associated with either current or past dog ownership [Motor Fitness Scale (*p* < 0.001), interaction with neighbors (*p* < 0.001), trust in neighbors (*p* < 0.001), social isolation (*p* < 0.001)] and cat ownership [interaction with neighbors (*p* < 0.001), trust in neighbors (*p* < 0.005), social isolation (*p* < 0.002)] (19).

The study concerned with incident frailty (*N* = 6,197) found greater reduced risks among past dog/cat owners (OR = 0.85, CI: 0.71–0.99, *p* < 0.05) than current (OR = 0.90, CI: 0.72–1.13) or never (OR = 1) owners (18). When separating dog from cat owners, past dog owners were significantly less likely to experience incident frailty (OR = 0.84, CI: 0.70–0.99, *p* < 0.05) than never (OR = 1) or current (OR = 0.86, CI: 0.65–1.13) dog owners. No statistically significant associations were found between incident frailty and cat ownership, but past cat owners (OR = 0.89, CI: 0.70–1.12) experienced slightly lower incident frailty risks than never (OR = 1) or current (OR = 1.04, CI: 0.77–1.40) owners. Compared to those who never owned a dog/cat, social function (i.e., interaction with neighbors) had a stronger negative association with incident frailty than those who currently or previously owned either pet species. They also found current dog/cat owners to be significantly younger than past and never owners and more often married [*p* < 0.001] with higher education [*p* < 0.001] and income rates [*p* < 0.001], obtained higher Motor Fitness Scale scores [*p* < 0.001] and frequencies of going outside [*p* < 0.001], and interacted with neighbors more often [*p* < 0.003] than past or never owners (18).

While the systematic review reported no statistically significant differences in frailty percentages among never, past, and current dog or cat owners, calculated odds ratios across the three included studies suggest that cat and/or dog ownership may be associated with benefits in the physical, psychological, and social health domains, which constitutes frailty (14). Additionally, an article concerned with psychological frailty (i.e., depressed mood and phenotypic frailty) (21) found the risks to be 40% lower for participants rearing grandchildren or pets (OR = 0.60, 95% CI: 0.47–0.76, *p* < 0.001) than those who did not (14, p. 4).

Finally, significant differences in cognitive frailty risk were uncovered among those (*N* = 2,638) who experienced social loss and did or did not own pets (4). Regardless of gender, experiencing either form of social loss had higher cognitive frailty rates than those who did not, but women with social loss and did not own a pet experienced the highest risk (15.5%) (OR = 2.06, 95% CI: 1.20–3.54, *p* < 0.01), followed by men with social loss who *did* own a pet (13.5%) (OR = 4.20, CI: 1.38–12.77, *p* < 0.05) (4). Conversely, rates were lowest among those who did not experience social loss and did own a pet [women: OR = 1.63 (CI: 0.99–2.71); men: OR = 1.50 (CI: 0.72–3.11)] (4). Overall, the prevalence of cognitive frailty was higher for women (9.3%) than for men (6.1%), suggesting sex differences may be at play and warrant consideration. Those deemed cognitively frail were older [*p* < 0.001], had lower education [*p* < 0.001] and income [*p* < 0.001] rates, higher psychological distress scores [*p* < 0.001], did not drink alcohol [*p* < 0.01], and did not own a pet [*p* < 0.01] (4).

## 4. Discussion

To the best of our knowledge, this is the first scoping review to explore the influence HAIs may have on multidimensional frailty for 50+ adults. While only four articles are included, thematic analysis ensued despite this restriction and yielded three overarching constructs.

### 4.1. Interconnected health effects of pet ownership

Physical, psychological, cognitive, and social health benefits can result from PO (4, 18, 25, 26) and yet, how these health domains affect one another are rarely assessed through the interdisciplinary perspectives of gerontology and anthrozoology. Each included article spoke to benefits in the physical, psychological, and social domains, but only one spoke to the influence of PO on cognitive health. Understanding the directionality behind the interdependent effects of these health domains is bound to result in more appropriate instrumentation and interventions regarding this incipient topic (27). One article spoke to social frailty presaging physical frailty (18). Another argued that dog ownership is associated with greater degrees of walking and social functioning, suggesting physical activity can promote social interaction (19). Relatedly, poor social health can result in social isolation, consequently impeding psychological health. The conceptualizations of cognitive frailty (4) and psychological frailty (14) build this argument further.

One article examining frailty differences among men and women spoke to the possibility of social roles, like gender norms, being influential to differences in cognitive frailty rates (4), further highlighting the interconnectedness of psychological, social, cognitive, and physical health. Cultural differences, including but not limited to social norms, warrant increased attention to better understand how these concepts might influence individual health domains and, consequently, frailty risk.

### 4.2. Current and past pet ownership as a protective factor

Evidence of PO as a protective mechanism against frailty was found, with dog ownership possibly having more influence than cat ownership. This may be because current and past dog owners reported greater degrees of physical activity *via* walking compared to those who never owned a dog (19). Or this finding could be due to the phenomenon known as the “pet ownership effect,” which argues that physically active individuals are more likely to own a pet than those who are not (18). Despite these causality concerns, this work argues that older adults who never owned a pet are consistently more likely to be frail than those who currently or previously owned a pet (18, 19). The varied effectiveness of past and current dog/cat ownership on frailty risk should be further explored.

### 4.3. Increased meaning and purpose through pet ownership

This work supports the notion that PO can result in increased meaning and purpose, which can positively influence psychological health (26, 28). Experiencing a sense of meaning and purpose in life is similarly associated with lower risks of ill-being (29). This is especially true for older adults with low socioeconomic status or weak social networks (28). The included review (14) revealed psychological frailty as significantly less prevalent in older individuals who rear either grandchildren or pets (21). Feeling intrinsically valued and purposeful through PO can afford “social situatedness” to older adults even in light of fluctuating autonomy (28). Interventions to increase meaning and purpose in older individuals should be developed and assessed for appropriateness and effectiveness.

### 4.4. Limitations

Meta-analysis was unattainable due to the inconsistent collection of frailty-based variables. Although we aimed to synthesize the largest body of literature possible, only incorporating articles published in English likely limited the number of hits, particularly considering the limited geographical diversity identified in this review. The generalizability of these findings is further tethered by all articles pertaining to older individuals in East Asia, predominantly Japan, potentially limiting the applicability of these findings to other older adult populations.

Varied findings across the included articles necessitate investigation into the heterogeneity of effects that PO may have on frailty for 50+ adults. Further, whether said associations are direct or indirect, and the specific mechanisms by which older adults benefit from HAIs regarding frailty risk remain unclear. Improved development and assessment of relevant studies and interventions may determine whether (and which) HAIs are successful in averting frailty in older adults, what health domains are affected when, and the directionality between any interconnected health effects.

### 4.5. Future directions

Future investigation should consider longitudinal analysis, companion animals outside of dogs and cats, and HAI studies and interventions outside of PO. Other studies and interventions that could glean meaning and purpose for older adults should be explored, like interacting with visiting animals or caring for houseplants.

More research is needed on PO in various housing environments. While most prefer to age in place (28), retirement community enrollment will likely increase as global societies continue aging. Related housing policies that prevent PO for older adults—in and outside of retirement communities—require review to ensure these regulations foster autonomy, avoid ageist and ableist language and intentions, and to eliminate the possibility of one choosing between housing or their pet (28). When older adults are better supported at the individual, communal, and policy levels to age in place the beneficial effects of PO are likely to ensue, including increased degrees of well-being and QOL (26).

Social determinants of health, like one’s built environment, are known to significantly impact QOL (30). These multifaceted health determinants likely contribute to frailty risk over time but are not yet adequately integrated into frailty assessments. Future research should also integrate the environmental health domain when assessing and defining multidimensional frailty.

## 5. Conclusion

We reported outcomes associated with HAI studies and multidimensional frailty in 60+ older adults. The importance of identifying effective frailty interventions cannot be understated, as such may be integral to upholding individual autonomy, well-being, and QOL throughout the lifespan. While only a few relevant HAI studies exist, synthesized findings suggest PO has interconnected and protective health effects for older adults, including heightened feelings of meaning and purpose.

Researchers could behoove current and future older adult populations by developing explicit definitions, instruments, and interventions that encompass the interconnected health variables related to frailty. Policymakers could suggest housing guidelines that prioritize the human-animal bond between older adults and their animal companions. Moving forward, interdisciplinary experts should reach consensus on a multidimensional frailty definition and appropriate HAI interventions for older adults as this often-overlooked population continues to expand worldwide.

## Author contributions

AT and KM administered the project and led the conceptualization as well as the design of this review. All articles were searched, charted, and assessed for eligibility by AT and MC. Thematic analysis and its validation were completed by AT, MC, KW, and KM. AT and MC wrote the original first draft, and AT took the lead on revisions. All authors contributed to the article and approved the submitted version.

## Conflict of interest

The authors declare that the research was conducted in the absence of any commercial or financial relationships that could be construed as a potential conflict of interest.

## Publisher’s note

All claims expressed in this article are solely those of the authors and do not necessarily represent those of their affiliated organizations, or those of the publisher, the editors and the reviewers. Any product that may be evaluated in this article, or claim that may be made by its manufacturer, is not guaranteed or endorsed by the publisher.

## References

[1] Walston J Bandeen-Roche K Buta B Bergman H Gill TM Morley JE. Moving frailty toward clinical practice: NIA intramural frailty science symposium summary. J Am Geriatr Soc. (2019) 67:1559–64. doi: 10.1111/jgs.15928, PMID: 31045254 PMC6830521

[2] Paulson D Lichtenberg PA. The Paulson-Lichtenberg frailty index: evidence for a self-report measure of frailty. Aging Ment Health. (2015) 19:892–901. doi: 10.1080/13607863.2014.986645, PMID: 25537004 PMC4480217

[3] Fried LP Tangen CM Walston J Newman AB Hirsch C Gottdiener J. Frailty in older adults: evidence for a phenotype. J Gerontol A Biol Sci. (2001) 56:M146–57. doi: 10.1093/gerona/56.3.M146 11253156

[4] Zhang S Wang Q Wang X Qi K Zhou Y Zhou C. Pet ownership and cognitive frailty among Chinese rural older adults who experienced a social loss: is there a sex difference? Soc Sci Med. (2022) 305:115100. doi: 10.1016/j.socscimed.2022.115100, PMID: 35690032

[5] Murman DL. The impact of age on cognition. Semin Hear. (2015) 36:111–21. doi: 10.1055/s-0035-1555115, PMID: 27516712 PMC4906299

[6] Sánchez-García S García-Peña C Ramírez-García E Moreno-Tamayo K Cantú-Quintanilla GR. Decreased autonomy in community-dwelling older adults. Clin interventions. Aging. (2019) 14:2041–53. doi: 10.2147/CIA.S225479, PMID: 31819386 PMC6873968

[7] Centers for Disease Control and Prevention. Well-being concepts (2018). Available at: https://www.cdc.gov/hrqol/wellbeing.htm#eight

[8] Virués-Ortega J Buela-Casal G. Psychophysiological effects of human-animal interaction: theoretical issues and long-term interaction effects. J Nerv Ment Dis. (2006) 194:52–7. doi: 10.1097/01.nmd.0000195354.03653.63, PMID: 16462556

[9] Garrity TF Stallones LF Marx MB Johnson TP. Pet ownership and attachment as supportive factors in the health of the elderly. Anthrozoös. (1989) 3 Available at: https://doi-org.du.idm.oclc.org/10.2752/089279390787057829

[10] Costa SPD. (2021) Daily mobility and social interaction of older adults’ dog owners: a scoping review. [dissertation] Coimbra (PT): Universidade de Coimbra, 41, 2609–2623, PMID: 36029015

[11] Keat KC Subramaniam P Ghazali SE Ami N. Review on benefits of owning companion dogs among older adults. Mediterr J Soc Sci. (2016) 7:4. doi: 10.5901/mjss.2016.v7n4p397

[12] Hughes MJ Verreynne ML Harpur P Pachana NA. Companion animals and health in older populations: a systematic review. Clin Gerontol. (2020) 43:365–77. doi: 10.1080/07317115.2019.1650863, PMID: 31423915

[13] Kruger KA Serpell JA. Animal-assisted interventions in mental health: definitions and theoretical foundations In: Fine AH, editor. Handbook on animal-assisted therapy. Third ed. Cambridge, MA: Academic Press (2010). 33–48.

[14] Kojima G Aoyama R Taniguchi Y. Associations between pet ownership and frailty: a systematic review. J Geriatr. (2020) 5:4. doi: 10.3390/geriatrics5040089, PMID: 33182245 PMC7709675

[15] Arksey H O’Malley L. Scoping studies: towards a methodological framework. Int J Soc Res. (2005) 8:19–32. doi: 10.1080/1364557032000119616

[16] Tricco AC Lillie E Zarin W O’Brien KK Colquhoun H Levac D. PRISMA extension for scoping reviews (PRISMA-ScR): checklist and explanation. Ann Intern Med. (2018) 169:467–73. doi: 10.7326/M18-0850, PMID: 30178033

[17] Faller JW Pereira DDN de Souza S Nampo FK Orlandi FDS Matumoto S. Instruments for the detection of frailty syndrome in older adults: a systematic review. PLoS One. (2019) 14:e0216166. doi: 10.1371/journal.pone.0216166, PMID: 31034516 PMC6488093

[18] Taniguchi Y Seino S Nishi M Tomine Y Tanaka I Yokoyama Y. Association of dog and cat ownership with incident frailty among community-dwelling elderly Japanese. Sci Rep. (2019) 9:1. doi: 10.1038/s41598-019-54955-9, PMID: 31819092 PMC6901519

[19] Taniguchi Y Seino S Nishi M Tomine Y Tanaka I Yokoyama Y. Association physical, social, and psychological characteristics of community-dwelling elderly Japanese dog and cat owners. PLoS One. (2018) 13:11. doi: 10.1371/journal.pone.0206399, PMID: 30427858 PMC6241120

[20] De Witte N Gobbens R De Donder L Dury S Buffel T Verte D. Validation of the comprehensive frailty assessment instrument against the Tilburg frailty indicator. Eur Geriatr Med. (2013) 4:248–54. doi: 10.1016/j.eurger.2013.03.001 23608069

[21] Shimada H Lee S Doi T Bae S Tsutsumimoto K Arai H. Prevalence of psychological frailty in Japan: NCGG-SGS as a Japanese national cohort study. J Clin Med. (2019) 8:1554. doi: 10.3390/jcm8101554, PMID: 31569684 PMC6832757

[22] Hwang HS Yoon JL Park BJ Choi HR Kwon IS Shinkai S. The validity and reliability of the Kaigo-Yobo checklist in Korean elderly. J Korean Gerontol Nurs. (2012) 16:121–32. doi: 10.4235/jkgs.2012.16.3.121

[23] Fried LP Borhani NO Enright P Furberg CD Gardin JM Kronmal RA. The cardiovascular health study: design and rationale. Ann Epidemiol. (1991) 1:263–76. doi: 10.1016/1047-2797(91)90005-W 1669507

[24] Folstein MF Folstein SE McHugh PR. “Mini-mental state”. A practical method for grading the cognitive state of patients for the clinician. J Psychiatr Res. (1975) 12:189–98. doi: 10.1016/0022-3956(75)90026-6, PMID: 1202204

[25] Friedmann E Gee NR Simonsick EM Studenski S Resnick B Barr E. Pet ownership patterns and successful aging outcomes in community dwelling older adults. Front Vet Sci. (2020) 7:7. doi: 10.3389/fvets.2020.00293, PMID: 32671105 PMC7330097

[26] Obradović N Lagueux É Latulippe K Provencher V. Understanding the benefits, challenges, and the role of pet ownership in the daily lives of community-dwelling older adults: a case study. Animals. (2021) 11:9. doi: 10.3390/ani11092628, PMID: 34573595 PMC8468022

[27] Gee NR Mueller MK Curl AL. Human–animal interaction and older adults: an overview. Front Psychol. (2017) 8:8. doi: 10.3389/fpsyg.2017.01416, PMID: 28878713 PMC5573436

[28] Toohey AM Hewson JA Adams CL Rock MJ. When ‘places’ include pets: broadening the scope of relational approaches to promoting aging-in-place. J Sociol Soc Welf. (2017) 44:107. doi: 10.1017/S0714980818000107

[29] Weziak-Bialowolska D Bialowolski P. Bidirectional associations between meaning in life and the health, emotional ill-being and daily life functioning outcomes among older adults. Psychol Health. (2022):1–17. doi: 10.1080/08870446.2022.2105842, PMID: 35903904

[30] Healthy People (2030). Social determinants of health Available at: https://health.gov/healthypeople/priority-areas/social-determinants-health

