# Corrigendum: Examining human-animal interactions and their effect on multidimensional frailty in later life: a scoping review

**DOI:** 10.3389/fpubh.2023.1258165

**Published:** 2023-08-01

**Authors:** Ashley Taeckens, Mary Corcoran, Kaipeng Wang, Kevin N. Morris

**Affiliations:** ^1^Institute for Human-Animal Connection, Graduate School of Social Work, University of Denver, Denver, CO, United States; ^2^Graduate School of Social Work, University of Denver, Denver, CO, United States

**Keywords:** frailty, human-animal interaction (HAI), pet ownership, older adults, health domains, quality of life

In the published article, there was an error in affiliation(s) [1]. Instead of “Institute for Human-Animal Interaction”, it should be “Institute for Human-Animal Connection.”

The authors apologize for this error and state that this does not change the scientific conclusions of the article in any way. The original article has been updated.

