# Supplementary material for: Examining human-animal interactions and their effect on multidimensional frailty in later life: a scoping review
Source: Front Public Health. 2023 Jun 21;11:1214127. doi: 10.3389/fpubh.2023.1214127 (PMC10321594; doi:10.3389/fpubh.2023.1214127)
Supplement: Supplementary file 1 [file Data_Sheet_1.PDF]

Supplementary Material

1 Supplementary Tables

Table 1a. Scoping review search terms on human-animal interaction studies regarding frailty.

Category	Search terms
Older adults§	(“older adult*” OR “old* people” OR “old* person” OR old OR “old* age*” OR “old old” OR “young old” OR “oldest old” OR Elder* OR Senior* OR “middle age*” OR Midlife OR “Late* life” OR “Life course” OR Ageing OR Aging OR Aged OR geriatric OR “residents” OR patients OR gerontolog* OR “age related change”)
Frailty	(Frail* OR Prefrailty OR “pre-frailty” OR “frail* index*” OR “cumulative deficit” OR “deficit accumulation” OR “accumulate* deficit” OR “intrinsic capacit*”)
Human-animal interactions	(“human-animal interaction*” OR “animal interaction*” or “human-animal” OR “human-canine*” OR “human dog” OR “dog companion*” OR “canine companion*” OR “companion dog*” OR “dog owner*” OR “service dog*” OR “guide dog*” OR “therapy dog*” OR “pet dog*” OR “dog walk*” OR Dog* OR Canine* OR “cat owner*” OR Cat* OR feline* OR “pet owner*” OR “pet companion*” OR Pet* OR “pet animal” OR “pet therapy” OR “human-pet” OR “pet keeping” OR “pet caring” OR “pet parenting” OR “cross-species parenting” OR “inter-animal species attachment” OR “animal owner*” OR “companion animal*” OR “animal companion*” OR “domesticated animal*” OR “domesticated pet” OR equine OR horse OR hedgehog OR amphibian OR turtle OR tortoise OR rabbit OR ferret OR “guinea pig” OR hamster OR “stick insect” OR Fish OR Fishing OR “small mammal” OR “exotic pets” OR Birds OR Reptiles OR parrots OR “therapy animal*” OR “service animal*” OR “support animal*” OR “resident animal*” OR “visiting animal*” OR “animal-assisted” OR “wildlife-assisted” OR “nature-assisted” OR “nature-based”)
NOT¥	(amyloid OR “cat scan” OR “Computed tomography scan” OR “CT scan*” OR “primary endocrine therap*” OR “PET scan*” OR “PET level*” OR tau OR “PET amyloid” OR child* OR “young children” OR “young adults” OR youth)

§ PubMed (includes MEDLINE) does not allow for truncation or the use of “*” to filter for variations of root words. The same terms were instead applied without this specifier.

¥ The NOT filter does not work for, and therefore was not applied to the search conducted in HABRI.

Table 1b. Attributes of included articles.

Reference	Age Range	Average Age	Sample Size	% Women	Frailty Concept	Location	Human-Animal Intervention	Study Design	Instrumentation
Zhang et al. (2022)	60-84	74.2	N=2,638	63.8%	Cognitive frailty	China	Pet ownership (unspecified)	Structured Interviews	Frailty Phenotype; MMSE; ADL Scale; K10
Kojima et al. (2020) ‡	60+	72.7	N=3	52.9%	Frailty	Japan#	Pet ownership (varies)	Systematic Review	n=2 KYCL-15, n=1 mCHS
Taniguchi et al. (2019)	65+	72.6	N=6,197	53.6%	Incident frailty	Japan	Pet ownership (dog and cat)	Cross-sequential (2YR)	KYCL-15, GDS
Taniguchi et al. (2018)	65+	N/A	N=11,233	52.3%	Frailty	Japan	Pet ownership (dog and cat)	Cross-sectional	KYCL-15, IPAQ-SF, MFS, TMIG-IC, WHO-5

# = All articles in this review were conducted in Japan, as well as the systematic review itself.

‡ This publication is a systematic review, and the provided results are averaged regarding the articles included in that study.

The following instruments were used in the reported studies [and measured]: ADL Scale: Activities of Daily Living Scale [functional and nutritional status/impairment]; GDS: Geriatric Depression Scale [depression]; IPAQ-SF: International Physical Activity Questionnaires-Short Form [physical activity]; K10: Kessler Psychological Distress Scale [psychological distress]; KYCL-15: Kaigo-Yobo Checklist 15 [frailty status]; mCHS: modified Cardiovascular Health Study [frailty status]; MFS: Motor Fitness Scale [basic motor ability]; MMSE: Mini-Mental Status Exam [cognitive impairment]. TMIG-IC: Tokyo Metropolitan Institute of Gerontology Index of Competence [functional capacity]; WHO-5: World Health Organization Five Well-Being Index [mental well-being].
